# Short-Term Cyclosporin A Treatment Reduced Serum Neurofilament-Light Levels in Diffuse but Not Focal Traumatic Brain Injury in a Piglet Model

**DOI:** 10.3390/biomedicines13102547

**Published:** 2025-10-18

**Authors:** Colin M. Huber, Akshara D. Thakore, Anna Oeur, Susan S. Margulies

**Affiliations:** Wallace H. Coulter Department of Biomedical Engineering, Georgia Institute of Technology and Emory University, Atlanta, GA 30332, USA; colin.michael.huber@emory.edu (C.M.H.); akshara.dakshesh.thakore@emory.edu (A.D.T.); anna.oeur@emory.edu (A.O.)

**Keywords:** traumatic brain injury, pediatric, serum biomarkers, cyclosporin A, swine

## Abstract

**Background/Objectives**: Traumatic brain injury (TBI) in the pediatric patient results in acute neurophysiological deficits and can have potential long-term sequelae, impacting neurodevelopment. Serum biomarkers are an active area of study for TBI prognosis and diagnosis. Cyclosporin A (CsA), an immunosuppressant drug with neuroprotective qualities, targets mitochondria to stabilize the neurometabolic energy crisis following TBI. The objective of this study was to determine the acute effect of CsA treatment following focal and diffuse TBI on piglet serum biomarkers associated with glial neurofilaments, axonal dysfunction, and neuronal injury. **Methods**: Biomarker concentrations of GFAP, Nf-L, and UCH-L1 were quantified retrospectively from porcine serum samples (*n* = 488) at multiple timepoints from three experimental groups: anesthetized sham (*n* = 10), controlled cortical impact (CCI, *n* = 49), or rapid, non-impact rotations (RNR, *n* = 151) of the head. Injured animals received 24 h post-injury intravenous administration of saline or one of four CsA treatment doses (10, 20, 40, or 60 mg/kg/day), and then, were sacrificed. **Results**: After RNR, GFAP levels significantly increased from baseline at 1 h and recovered by 1 day to healthy reference ranges, while Nf-L increased at 1 day. Multiple CsA treatment doses (10, 40 mg/kg/day) significantly reduced Nf-L levels at 1 day compared to the untreated group. After CCI, GFAP and Nf-L increased at 1 day; there were no significant treatment effects. **Conclusions**: Focal and diffuse brain injury mechanisms resulted in distinct biomarker timelines. CsA reduced Nf-L levels at 1 day after diffuse TBI, showing promise of acute therapeutic benefit and warranting further investigation in extended timelines.

## 1. Introduction

Traumatic brain injury (TBI) is a consequential injury frequently caused by motor vehicle accidents and collisions in contact sports causing acute and lingering neurophysiological deficits along with potential long-term sequelae [1,2,3]. Following severe TBI, patients are admitted to neurointensive care for multimodality neuromonitoring [4]. Immediate interventions typically focus on surgical intervention or clinical management to prevent mortality and increasing morbidity such as reducing intracranial pressure via fluid management, sedation, and/or craniotomy [5,6,7,8]. The heterogeneity of TBI mechanisms and symptom phenotypes make treatment especially challenging [6,8]. Pediatric patients are especially vulnerable to TBI because they have increased rates of injury and TBI is the leading cause of death in children [1,2,3]. TBI in the rapidly developing brain of pediatric patients could cause significant effects on neurodevelopment, and, therefore, there is an opportunity for therapeutic intervention applicable to all ages to provide substantial improvement to quality-adjusted life years. Numerous pharmaceutical interventions have shown promise in rodent models but failed to achieve neuroprotective benefits in human trials [9,10]. Particularly challenging in the translation from animal to human studies is the lack of heterogeneity of injury mechanism in animal studies, and the paucity of outcome metrics common to animals and humans. We posit that there is a critical need to evaluate treatments with noninvasive outcome metrics, such as blood-based biomarkers, to help evaluate interventions and translate from preclinical models of TBI to clinical trials.

Blood-based biomarkers have demonstrated diagnostic and prognostic utility for TBI in limited applications. In 2018, the Food and Drug Administration (FDA) approved plasma concentrations of glial fibrillary acidic protein (GFAP) and ubiquitin carboxyl-terminal hydrolase L1 (UCH-L1) measured in the first 12 h after injury to rule out the need for CT scans for hemorrhagic TBI in adults [11,12,13], and in 2024, whole blood GFAP and UCH-L1 measured within 24 h were approved to rule out CTs [14,15]. Initial studies of the same blood-based biomarkers within 6 h of injury in children performed effectively to differentiate patients with and without positive CTs [16,17]. GFAP and neurofilament-light (Nf-L) levels were elevated for up to 4 and 12 weeks, respectively, following a sports-related concussion, especially amongst patients who experienced loss of consciousness, and higher levels were predictive of longer times for return times to training [18]. From a large TBI study of adult patients, GFAP, UCH-L1, and Nf-L levels measured in the first 24 h after injury highly predicted prognosis of mortality and 6-month clinical outcomes [19,20].

Few studies have explored blood-based biomarkers’ ability to track therapeutic benefit. Following multiple TBI mechanisms (i.e., fluid percussion injury, controlled cortical impact (CCI), and penetrating ballistics-like brain injury) in rats, Levetiracetam treatment improved behavioral outcomes showing acute therapeutic benefit, and in parallel, lower serum levels of GFAP and phospho-neurofilament-H, a marker of axonal injury, were measured at 24 h compared to the vehicle group [21,22]. In pigs after CCI, serum GFAP decreased after treatment of Valproic acid aligning with decreased brain lesion volume [23], and initial studies in piglets indicated the capability of Nf-L in cerebrospinal fluid (CSF) to quantify the therapeutic benefit of Cyclosporin A (CsA) [24]. Therefore, multiple blood-based biomarkers have been successfully applied in preclinical studies beyond their FDA-approved diagnostic potential to measure therapeutic benefit and should be tested more extensively. Pigs provide a particularly compelling, well-established model for preclinical TBI studies [25,26,27] due to similarities in neuroanatomical structure with humans, including a gyrencephalic brain and comparable white to grey matter ratios, allowing accurate representation of injury biomechanics [28,29,30,31].

Following TBI, a neurometabolic cascade emerges due to disrupted ionic exchange, energy production, and neurotransmission leading to increased inflammation, oxidative stress, and neuronal cell death [32,33]. Mitochondria play a critical role in calcium (Ca^2+^) homeostasis; however, high Ca^2+^ sequestering can decrease ATP production and affect the mitochondrial permeability transition pore (mPTP), leading to the release of apoptotic and ROS into the cell [34,35]. The developing brain is especially sensitive to excitotoxicity [34]; therefore, stabilizing mitochondrial function is an important target for TBI treatment and recovery. CsA, a widely available anti-inflammatory medication used for immune suppression following transplant surgery [36], inhibits the opening of the mPTP and stabilizes the mitochondria following TBI [30,37,38,39]. CsA has a U-shaped response curve to dosage concentration for both mitochondrial pore function and clinical outcomes [37,40]. The U-shape is created by an effective stabilization of the mPTP at lower concentrations, but higher concentrations can cause toxicity by affecting the electron transport chain and subsequent energy production as well as increase in reactive oxygen species [41]. Further, continuous administration of CsA creates even pharmacokinetics for consistent concentration and therapeutic effect on the mPTP [42,43], highlighting the importance for CsA dosage choice. CsA is FDA-approved for all ages (including safe use in children) for multiple treatment applications including organ transplants, uveitis, and amyotrophic lateral sclerosis, and CsA has been safely administered in adult patients with severe TBI [44]. Acute CsA treatment (24 h) improved mitochondria function and axonal injury after CCI and rotational diffuse injury in piglets [30], and CsA preserved mitochondrial function, improved cerebral blood flow, and decreased brain injury volume following rotational diffuse injury in 3–5 day-old newborn piglets [45]. CsA reduced mitochondrial and axonal injury following closed-head weight drop injury in rats [46,47] and following focal and diffuse injury in piglets [30]. Similarly, CsA treatment reduced lesion volume following focal CCI with a dose-dependent response of high doses associated with smaller lesions [35,43]. Taken together, CsA administered after TBI has shown promising clinical treatment efficacy for multiple TBI mechanisms (i.e., focal and diffuse) across multiple animal models, and piglets provide an excellent model for development in the pediatric brain based on similar myelination timelines and white matter volume to humans [29,48].

In this study, we investigate noninvasive blood-based biomarkers as a useful metric to track therapeutic benefit acutely after two distinct forms of TBI in piglets, CCI and rapid, non-impact rotations (RNR). The first objective was to determine the effect of time and injury type on serum biomarker concentrations of glial, neuroaxonal, and neuronal injury—GFAP, Nf-L, and UCH-L1. A second objective was to determine the value of serum biomarkers as a marker for treatment efficacy [30,49], using acute (24 h) CsA treatment with demonstrated efficacy at multiple doses in reducing mitochondrial dysfunction and neuropathology as a use case [30]. The current study adds the use of blood samples and measurement of circulating biomarker levels to a prior study investigating the efficacy of 24 h CsA administration following TBI [30].

## 2. Materials and Methods

TBI biomarker concentrations were quantified retrospectively from porcine serum samples (*n* = 488), nearly all collected as part of a prior study designed to assess the short-term treatment efficacy of 24 h CsA administration following TBI, with the effects on neuropathology and mitochondrial function previously published [30]. The protocol was approved by the Institutional Animal Care and Use Committee of the University of Pennsylvania, where animal studies were performed.

### 2.1. Study Design

**Figure 1:**
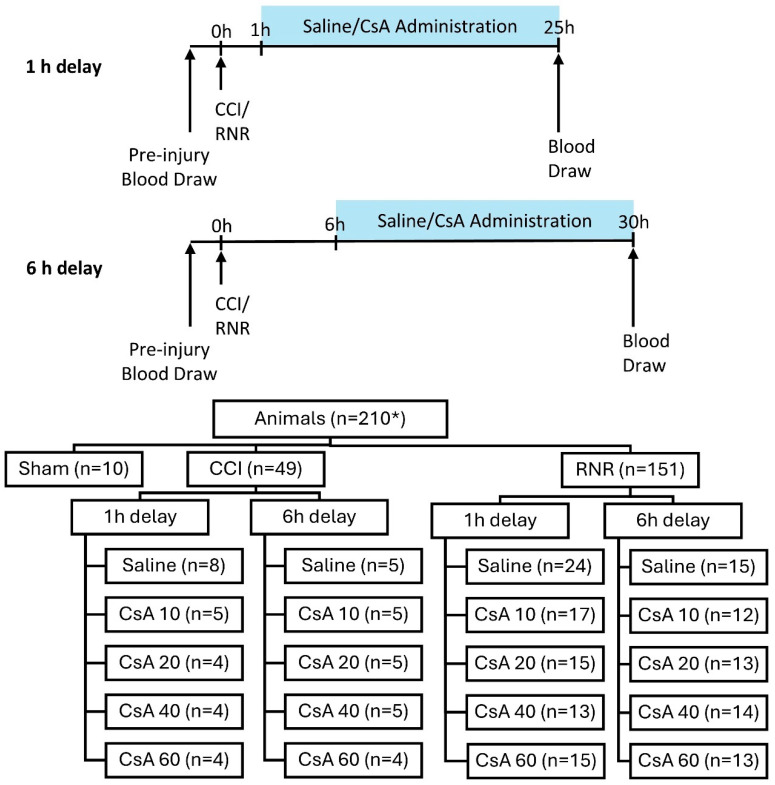
Experimental design of two treatment delay timings and distribution of animals to experimental groups. * *n* = 210 represents all animals that had serum samples available for selection.

Animal subjects (*n* = 210 with available serum samples) were allocated to one of three experimental procedures: anesthetized sham (*n* = 10), CCI (*n* = 49), or RNR (*n* = 151) of the head centered on the cervical spine (Figure 1). Injured animals were assigned to one of two treatment delays (IV administration beginning 1 h or 6 h post-injury), and either saline or one of four CsA treatment doses (10, 20, 40, or 60 mg/kg/day) for a total of 20 groups (2 injury types × 2 treatment delays × 5 treatment doses). Saline or CsA were administered continuously via an intravenous (IV) infusion pump to maintain target concentrations similar to clinical studies in humans [50]. The anesthetized sham group received neither saline nor CsA administration. Dosages were selected to cover the known effective and safe ranges using published dosing guidelines, prior clinical trials, and previous published data in infant piglets [45] and rodent neuro-injury [40,51,52], with appropriate allometric scaling.

### 2.2. Porcine Traumatic Brain Injury Models

A CCI model was designed in piglets to produce a focal injury via a blunt stainless-steel indenter tip directly contacting the cortical surface via craniotomy (i.e., hole in skull, with underlying dura retracted) of the right rostral gyrus, which is the somatosensory cortex associated with snout sensation [30]. The custom-designed device was mounted to the skull and a spring-loaded cylindrical tip (1.07 cm diameter) delivered a 5.9 mm indention over 4 ms [53].

A RNR head injury model was used to produce diffuse mild to moderate TBI in piglets following a single rotation (12–20 ms) in the sagittal plane, associated with bilateral widespread axonal injury and alterations in mitochondrial function [30]. Piglets’ snouts were securely attached to loading linkages driven by a custom HYGE pneumatic actuator device inertially loading the pig head for a controlled 60 degree rotation path [54]. Angular velocity data was captured using single-axis angular rate sensors in duplicate (Applied Technology Associates, Albuquerque, NM, USA) attached to the side arm of the RNR device. Angular velocity was measured at 10 kHz and filtered at collection using a CFC 1000 filter. For calculating angular acceleration, angular velocity was filtered with a CFC 180 filter, and a two-point derivation was completed [49]. Mean peak angular velocities and acceleration for RNR overall were 124.4 ± 4.6 rad/s and 53.4 ± 12.1 krad/s^2^, and there were no significant velocity differences for any of the treatment groups compared to saline via t-test (all *p* > 0.055).

### 2.3. Serum Sample Acquisition and Analysis

Whole blood was collected in all animals (*n* = 210) at baseline prior to injury or anesthesia and 1 day after injury (25/30 h depending on treatment delay). In addition, for sham and most 1 h delay CCI/RNR groups only (*n* = 71), whole blood was also collected 1 h after injury, prior to starting saline/CsA. Approximately 3 mL of whole blood was collected at each blood draw via the intravenous ear port used for saline/treatment administration. Based on serum availability, a total of 488 serum samples were collected from 210 four-week-old female Yorkshire piglets (Table 1). The whole blood was centrifuged, and serum was stored frozen at −80 °C. Serum was extracted and diluted at 4×; then, the Simoa Human Neurology 4-Plex A assay (Quanterix Corp., Billerica, MA, USA) was used to quantify GFAP, Nf-L, tau, and UCH-L1.

### 2.4. Data Processing

Serum samples were tested in duplicate and averaged. Samples from the same animals at different timepoints were tested on the same plate. Samples with a high coefficient of variance > 25% (CV = σ/μ * 100) were retested when possible or removed from further analysis.

For retests, predominately triggered for UCH-L1 measurements, the retest UCH-L1 value was used while the original GFAP and Nf-L were presented to minimize plate to plate variance. Only 3 total high CV readings (1 GFAP, 1 Nf-L, and 1 UCH-L1) failed retest and were removed from analysis.

As previously described [49] and aligning with established practice [55], samples that were determined to be below the Quanterix level of detection (<LOD) or the lower limit of quantification (<LLOQ) were assigned the batch-specific value of LOD/2 or LLOQ/2, respectively, for purposes of analysis and for visual displays. Similarly, samples with values greater than the Quanterix upper limit of quantification (>ULOQ) were assigned the batch value of the ULOQ. There were 8 datapoints for GFAP > ULOQ that were assigned values 2892–3276 pg/mL, respective to the ULOQ established on the measurement batch.

There were 11 baseline (t = 0) values (3 GFAP, 5 Nf-L, 3 UCH-L1) representing 9 subjects that were determined to be outliers, defined as greater than four standard deviations away from the group mean. All timepoints of that specific biomarker for those subjects were removed from analysis, while other biomarker data for these subjects were utilized.

After removing the samples described above, the number and percentage of samples that were <LLOQ and <LOD were quantified. The percentage of samples below LLOQ or LOD varied by biomarker: 0% GFAP, <1% Nf-L, and 36% UCH-L1. As anticipated from our personal communication with Quanterix and a prior study indicating the human tau antibody is non-reactive to swine [56], it was not surprising that a high percentage of tau measurements were <LLOQ (>74% of values in all timepoints). Therefore, total tau was not analyzed further in the current study.

### 2.5. Statistical Analysis

Our prior study of healthy female 4-week-old Yorkshire piglets established and validated 95% healthy porcine reference ranges (RR) for GFAP (6.3–69.4 pg/mL), Nf-L (9.5–67.2 pg/mL), and UCH-L1 (3.8–553.7 pg/mL) [49]. Serum biomarker data were compared to the healthy RRs within injury type, treatment dose, and timepoint using Fisher’s exact tests (*p* < 0.05). From two-sample t-tests of saline groups, it was determined that the 25 h and 30 h timepoints were not significantly different within injury type (all *p* > 0.259); therefore, 25 h and 30 h were combined into 1 day for analysis (Table 1). Two-way ANOVA was used to determine injury type differences (injury type × timepoint) in untreated animals. For treatment analysis, differences from baseline pre-injury were determined for each treatment dose within injury via Wilcoxon sign-rank test (*p* < 0.05), and treatment doses were compared to saline within injury using Mann–Whitney U tests (*p* < 0.0125 with Bonferroni correction).

## 3. Results

### 3.1. Acute Serum Biomarker Levels for Untreated Traumatic Brain Injury Types

For the 1 h delay groups, blood was drawn prior to the initiation of CsA administration, when there was no effect of treatment within injury type. Therefore, not surprisingly, we found no influence of group, saline group and any CsA treatment arms, for any biomarker when comparing 1 h to baseline using Mann–Whitney U tests (*p* > 0.096). Therefore, all groups were utilized to evaluate biomarker levels at baseline and 1 h.

**Figure 2:**
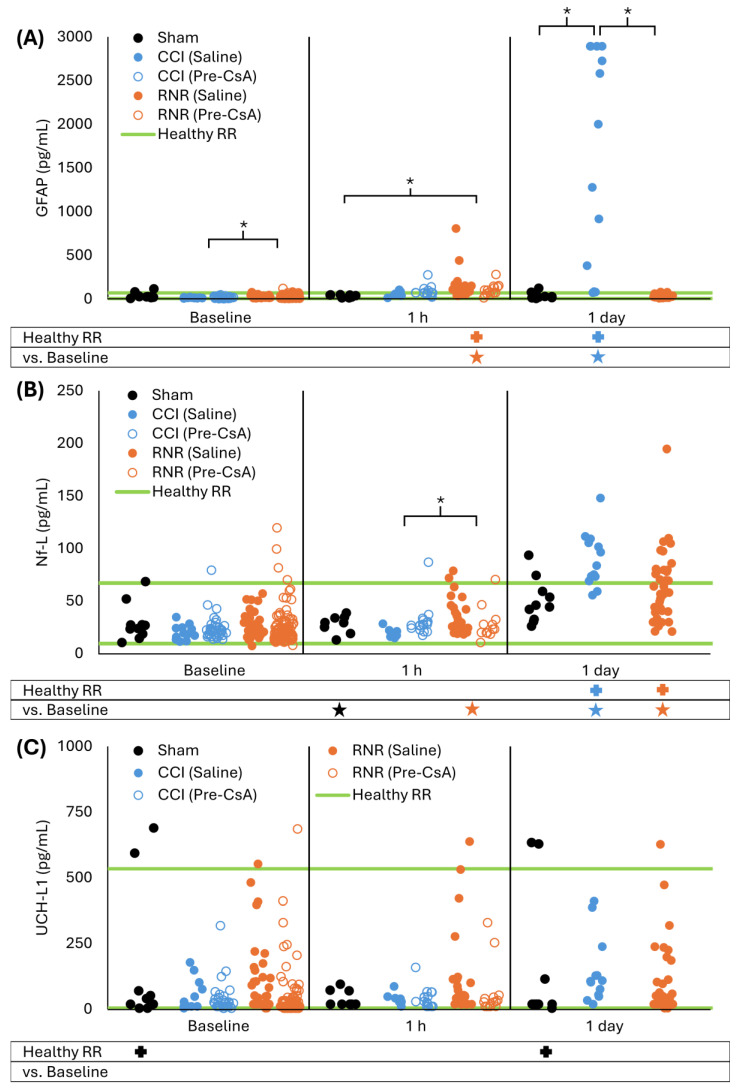
Distinct serum GFAP (**A**), Nf-L (**B**), and UCH-L1 (**C**) acute time-courses were observed following sham, focal controlled cortical impact, and diffuse rotational traumatic brain injury in piglets. * *p* < 0.05 two-way ANOVA comparisons to baseline within injury type (below graph) and between injury type within timepoint (within graph with brackets). + *p* < 0.05 difference from healthy RR. Symbol color corresponds to analyzed group for comparison to healthy RR or baseline.

For sham animals, serum GFAP levels remained unchanged 1 h and 1 day after anesthesia compared to baseline (*p* > 0.999) and remained within a previously established healthy reference range (RR) for 4-week-old piglets (all *p* > 0.125, Figure 2A) [49]. After CCI, untreated animals’ GFAP levels did not differ from sham animals or their baseline at 1 h (all *p* > 0.988) but were above the healthy RR (*p* < 0.001). At 1 day after CCI, GFAP levels rose significantly above the healthy RR and higher than baseline and sham (all *p* < 0.001). After RNR, GFAP levels at 1 h were significantly above the healthy RR (*p* < 0.001) but did not differ from their baseline or sham (all *p* > 0.301). In contrast to CCI, this increase was transient, and GFAP levels at 1 day after RNR returned to within the healthy RR and did not differ from their baseline or sham at 1 day (all *p* > 0.699). When completing a repeated measures ANOVA analysis for only saline animals, thus removing the baseline and 1 h data for CsA groups, the results did not meaningfully change. At baseline, RNR was greater than CCI (*p* = 0.014). At 1 h, RNR was greater than baseline and sham (all *p* < 0.011), and CCI at 1 h remained within the healthy RR (*p* = 0.065). The distinct temporal patterns of GFAP levels between CCI and RNR resulted in significantly different levels at 1 day.

For sham animals, serum Nf-L levels did not significantly differ from the healthy RR at baseline or 1 h and 1 day after anesthesia (*p* > 0.093, Figure 2B), and Nf-L levels were unchanged from baseline at 1 h and 1 day (all *p* > 0.230). After CCI, untreated animals’ Nf-L levels were unchanged at 1 h and were within the healthy RR (*p* > 0.994). However, like GFAP, 1 day after CCI, Nf-L levels at 1 day increased compared to baseline and sham at 1 day and were above the healthy RR (all *p* < 0.001). After RNR, untreated animals’ Nf-L levels at 1 h did not change from baseline, were not different from sham at 1 h, and remained within the healthy RR (all *p* > 0.229). However, unlike GFAP, at 1 day after RNR, Nf-L levels increased above the healthy RR and compared to baseline (both *p* < 0.001), but they did not differ from sham (*p* = 0.409) and were lower than after CCI (*p* = 0.002). For repeated measures ANOVA of only saline animals, sham Nf-L levels at 1 h were lower than baseline, and RNR at 1 h was significantly greater than baseline and CCI (all *p* < 0.035). At 1 day, CCI levels did not significantly differ from RNR or sham (*p* > 0.076). In summary for both CCI and RNR, Nf-L increased significantly in untreated animals at 1 day post-TBI.

Serum UCH-L1 levels showed high variability at baseline, as noted in our previous study reporting a wide healthy RR [49]. Serum UCH-L1 levels remained unchanged for sham animals compared to baseline at both 1 h and 1 day after anesthesia (*p* > 0.609, Figure 2C), but sham levels spread significantly outside the healthy RR at baseline and 1 day (both *p* = 0.034). For both CCI and RNR, levels remained unchanged compared to baseline at 1 h and 1 day (all *p* > 0.068). For CCI, UCH-L1 levels were within the healthy RR at all timepoints (all *p* > 0.060). For RNR, UCH-L1 levels were outside the healthy RR at baseline (*p* = 0.001) but remained within the healthy RR for 1 h and 1 day timepoints (*p* > 0.999). For repeated measures ANOVA, RNR UCH-L1 levels at baseline were within the healthy RR (*p* > 0.999). In summary, UCH-L1 in untreated piglets was unaffected in the first day after RNR and CCI, and, therefore, UCH-L1 did not warrant further analysis in our data because it was not sensitive for measuring treatment efficacy.

Taken together in untreated animals, after CCI, GFAP and Nf-L remained significantly outside the healthy RR at 1 day, and for RNR, Nf-L remained significantly outside the healthy RR at 1 day. Therefore, to assess 24 h of CsA treatment, we focused our analysis on GFAP and Nf-L at 1 day.

### 3.2. Effect of Acute Cyclosporin A Treatment on Serum Biomarkers

**Figure 3:**
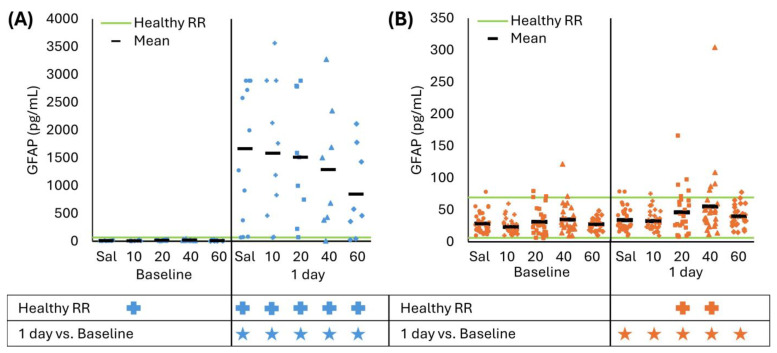
(**A**) At 1 day, CCI increased GFAP levels from baseline and healthy RR for saline and all CsA doses. Saline was not significantly different than any treatment dose. (**B**) RNR had slightly elevated GFAP levels at 1 day but within the healthy RR for saline and CsA 10 and 60 mg/kg/day doses; however, GFAP levels were above the healthy RR for 20 and 40 mg/kg/day doses. There were no significant effects for any treatment group compared to saline. * *p* < 0.05 comparison to baseline within injury. + *p* < 0.05 difference from healthy RR. Symbol color corresponds to analyzed group for comparison to healthy RR or baseline. Sal: saline.

For all CCI treatment groups, serum GFAP levels at 1 day were greater than baseline and the healthy RR (all *p* < 0.016), and none of the treatment doses significantly decreased levels compared to saline (all *p* > 0.059, Figure 3A). The baseline 10 mg/kg/day CsA group was below the healthy RR due to a few samples being below the lower RR limit (*p* = 0.022). For RNR, GFAP levels for all treatment doses at 1 day were greater than baseline (all *p* < 0.004), and CsA 20 and 40 mg/kg/day were significantly above the healthy RR (*p* < 0.029). As expected in RNR, there were no significant differences between the saline or treatment doses at baseline or 1 day (all *p* > 0.062). We conclude that GFAP was not sensitive to detect the effect of continuous treatment for 24 h, at any dose administered after either focal or diffuse TBI.

**Figure 4:**
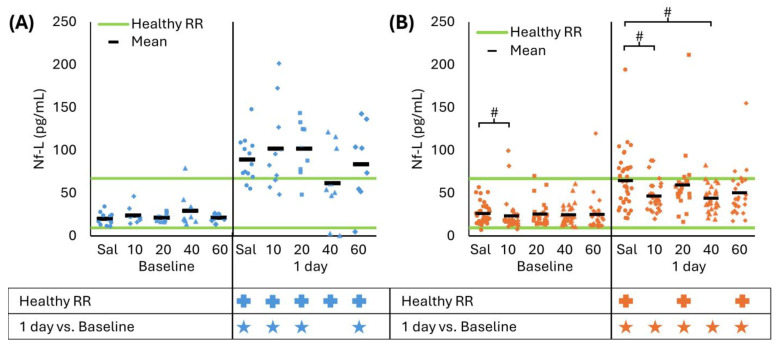
(**A**) CCI increased Nf-L levels compared to baseline and healthy RR at 1 day for saline, and all CsA doses except 40 mg/kg/day did not differ from baseline. There were no significant effects for any treatment dose compared to saline. (**B**) RNR had elevated GFAP levels at 1 day compared to baseline for all treatment doses, and saline, and CsA 20 and 40 were above the healthy RR. CsA 10 and 40 mg/kg/day doses had lower levels than saline at 1 day that were within the healthy RR. # *p* < 0.0125 comparison to saline within timepoint. * *p* < 0.05 comparison to baseline within injury. + *p* < 0.05 difference from healthy RR. Symbol color corresponds to analyzed group for comparison to healthy RR or baseline. Sal: saline.

Nf-L revealed a treatment effect at 40 mg/kg/day for both CCI and RNR and at 10 mg/kg/day for RNR only. Specifically, after CCI, all CsA treatment doses had Nf-L levels above the healthy RR at 1 day (all *p* < 0.001), but CsA 40 mg/kg/day had Nf-L levels that were indistinguishable from baseline (*p* = 0.098) while all other doses were greater than baseline (all *p* < 0.016, Figure 4A). However, none of the treatment doses resulted in Nf-L that differed from untreated CCI (all *p* > 0.062). For RNR at baseline, Nf-L levels for the CsA 10 mg/kg/day dose were greater than saline (*p* = 0.009). For CsA 10 and 40 mg/kg/day, Nf-L levels were greater than baseline (all *p* < 0.001); however, levels indicated therapeutic benefit by remaining within the healthy RR (*p* > 0.070) and were significantly lower than saline (*p* < 0.010). In summary, using Nf-L as a biomarker of therapeutic benefit, multiple CsA doses indicated a significant treatment effect at 24 h. Specifically, the RNR 10 and 40 mg/kg/day group had levels that returned to the healthy RR and were significantly lower than untreated RNR, and the CCI 40 mg/kg/day group resulted in levels remaining close to baseline.

There was a weak correlation (R^2^ < 0.155) between serum biomarker levels at 1 day and clinical measures of mitochondrial function and neuropathology (axonal injury + ischemic neuronal injury). These weak linear trends were unsurprising because there are only loose indirect relationships between these clinical measurements and serum biomarker levels, and this was a cohort of convenience without all samples available.

## 4. Discussion

TBI is a consequential injury, especially in the vulnerable pediatric population, causing acute neurophysiological deficits and potential long-term sequelae. The first objective of this study was to determine the effect of time and injury type on serum biomarker concentration. Because there is a paucity of available clinical treatments for TBI, the second objective of the current study was to determine the value of serum biomarkers as a marker for treatment efficacy [30,49], using CsA treatment with demonstrated efficacy at multiple doses in reducing mitochondrial dysfunction and neuropathology as a use case [30]. In this study, we report distinct biomarker timelines and magnitude changes after focal and diffuse TBIs with persistent elevation of GFAP (focal only) and Nf-L (focal and diffuse TBI) at 1 day. After a 24 h CsA infusion, Nf-L was significantly reduced for 10 and 40 mg/kg/day for RNR diffuse injury.

Our results show an influence of time, injury type, and their interaction for GFAP and Nf-L. For GFAP, untreated RNR animals had an immediate (<1 h) rise in GFAP and recovered to baseline by 1 day after injury, while untreated CCI animals saw a continuing increase at 1 day after injury. For Nf-L, both CCI and RNR increased by 1 day after injury, but CCI levels were a greater magnitude than RNR. UCH-L1 remained unchanged for both CCI and RNR.

Serum GFAP, a marker of glial injury, is also connected to blood–brain barrier (BBB) disruption [56]. GFAP production is ramped up during astrogliosis [57], and astrogliosis is centered around areas of blood-based proteins entering the brain following TBI, creating an access point for GFAP to enter into the circulatory system [58,59]. Combined, serum GFAP serves as an effective detector of hemorrhages in the brain [15], and currently, the FDA has approved the use of GFAP and UCH-L1 biomarkers in TBI in a limited scope and timeframe (<24 h after injury) to rule out a CT scan commonly used to identify CT+ lesions [12,60]. GFAP has also shown excellent performance in detecting positive CT results within 30 and 60 min post-injury and prognostic potential for 1-week outcomes [61]. However, based on the current study, there are important differences in biomarker timelines based on the underlying injury, which affects both the interpretation of biomarker levels (i.e., severity within a mechanism) and may influence monitoring and treatment decisions based on if prognosis is expected to worsen. A prior study in humans found that the worst CT scans for traumatic subarachnoid hemorrhages were typically observed from 12 to 24 h after injury and suggested repeat CTs to monitor hemorrhage development [62]. In the current study, CCI had a delayed GFAP increase compared to RNR and peaking 1–2 days after CCI in a prior study [24]. Further, a TRACK-TBI study found effective discrimination of healthy controls, CT+, and CT- patients using GFAP levels up to 3 days post-injury [63]. Continued monitoring of biomarkers > 24 h for some brain injury mechanisms may be considered when initial biomarker levels did not meet criteria for a CT scan to monitor for slower developing brain bleeds that may require surgical intervention. Therefore, biomarkers may have further utility in injury phenotyping and mechanism-specific care beyond currently approved applications.

Serum Nf-L serves as an effective marker for neuroaxonal injury representative of white matter injuries in humans and is highly correlated with CSF Nf-L levels independent of BBB integrity, which recovers over time after TBI [64,65,66,67,68]. In the current study, Nf-L levels higher 1 day after CCI (89.4 ± 7.2 pg/mL) were compared to RNR (64.9 ± 5.6 pg/mL). This result aligned with greater total injured area of axonal injury plus infarct/ischemic lesions after CCI (~3.5%) compared to RNR (~1%) in the original base study cohorts [30]. Although the CCI was focal, it created a large, concentrated lesion causing substantial neuronal and axonal injury [30]. Therefore, we expected serum Nf-L levels to correlate with neuroaxonal injury levels after TBI and serve as a therapeutic measure for CsA to mitigate injury after TBI. Nf-L levels typically peak around 1-week post-injury in pigs [24,49,69], so these initial increases are likely to continue for injured animals. Treatment < 24 h may provide temporary improvements or change the trajectory of Nf-L levels for greater treatment efficacy at later timepoints. After CCI in pigs, continuous CsA treatment for 5 days showed a trend towards reduced Nf-L levels compared to a placebo group starting at 5 days post-injury [24]. In general, serum Nf-L levels correspond to TBI severity and are prognostic of recovery and clinical outcomes [70,71], and Nf-L levels even within the first 24 h and/or at hospital admission are predictive of mortality and clinical recovery [20,72]. Therefore, reductions in acute Nf-L levels and axonal injury might be expected to predict improved short- and long-term outcomes, and there are several recent and ongoing clinical trials employing Nf-L levels to measure treatment efficacy (e.g., NCT06790095, NCT04418440, NCT03345550, and NCT06163833).

In the current study, UCH-L1, a marker of neuronal injury, did not differ 1 h or 1 day after CCI or RNR injury compared to baseline, sham, or the healthy RR [73]. Similarly, our prior study of porcine RNR injuries did not result in UCH-L1 level changes 30 min, 1 h, 6 h, 1 day, or 1 week after injury [49], despite identifying intracranial hemorrhage 3–8 h and 5–6 days after injury [74]. As previously stated, baseline UCH-L1 levels had higher variability and ranges than previously reported in human subjects [49], making UCH-L1 a more challenging biomarker to elicit diagnostic criteria. The lack of UCH-L1 increase after injury in our porcine focal and diffuse TBI models hampered the use of UCH-L1 to track recovery or measure therapeutic benefit. The known rapid rise in UCH-L1 following severe brain injury in humans and distinction between those with and without intracranial hemorrhage on CT may allow utility [11,12], and, therefore, human measurement and evaluation should be pursued.

CsA’s mechanism of action stabilizes the mitochondrial mPTP to mitigate the Ca^2+^-induced proteolytic cascade [46,47] and improve ATP production [45], which, combined effectively, reduces neuronal and axonal injury after TBI in immature and adult animal models [45,46,47,75]. Further, CsA has shown promise in clinical trials for safe use [50,76] and improved clinical outcomes following severe traumatic brain injuries in adults [42,44]. Hatton et al. observed improved 6-month clinical function outcomes for those receiving CsA administration compared to a placebo group [42], and Kelsen et al. observed decreases in GFAP, Nf-L, and UCH-L1 levels after 5 days of CsA administration compared to initial post-injury levels [44]. In the current study, Nf-L levels after RNR were lower than saline following 24 h CsA administration at 10 and 40 mg/kg/day, and Nf-L levels did not increase from baseline at 1 day after CCI when 40 mg/kg/day CsA was administered. Based on prior research indicating a U-shaped response curve for CsA [37,40], 60 mg/kg/day may have edged beyond the best therapeutic range [30]. Although 20 mg/kg/day was previously found to be therapeutic, our study could have been influenced by a small sample size, biological variability (higher variance in RNR 20/mg/kg/day group), and a small Nf-L signal at 24 h. Therefore, further studies investigating multiple dosages at longer survival times are recommended to determine the best treatment administration plan. In the current study, while there was no significant treatment effect of CsA on GFAP after CCI, there was a trend of increasing dose decreasing GFAP levels. Relatively small sample sizes and substantial variability across animals may have limited the study’s ability to detect significant changes. Further, CsA effects on GFAP may have occurred beyond the study limitation to 1 day post-injury because peak GFAP levels occur 1–3 days post-injury after CCI [24,63]. However, a few other preclinical studies have used GFAP to quantify acute therapeutic benefit. In rats after CCI, decreased levels of GFAP and phospho-neurofilament-H 24 h after Levetiracetam treatment showed acute therapeutic benefit [21,22]. In pigs after CCI, acute Valproic acid (<7 h after injury) decreased serum GFAP levels and brain lesion volume [23].

A few other preclinical studies have used blood-based Nf-L for therapeutic benefit. In a study of CCI in 4-week-old piglets, Nf-L continued increasing through 5 days, and 20 mg/kg/day CsA administration for 5 days showed significant trends of lowering Nf-L levels through the 5 days, indicating that extended CsA treatment may be beneficial [24]. Acute Valproic acid treatment (<7 h after injury) in pigs after CCI led to similar Nf-L levels that were similar to untreated animals, unsurprisingly, due to the delayed timeline of Nf-L changes after injury, which has greater utility at later timepoints [23]. In our study, Nf-L showed promise to track treatment efficacy following TBI, and acute CsA administration at multiple doses showed improved Nf-L levels. We recommend further investigation of acute and longer CsA treatment windows beyond 1 day post-injury and the use of serum Nf-L for therapeutic benefit in parallel and potentially translatable preclinical and clinical trials. Interestingly, there is a new clinical trial measuring the efficacy of Cyclosporin A treatment for 3 days post-injury and using GFAP, Nf-L, and UCH-L1 as outcome measures to evaluate treatment efficacy (NCT06790095).

### Limitations

There were several limitations to the current study. First, due to limited availability of serum, it was not possible to quantify biomarker levels for all animals from the original study centered on axonal injury and mitochondrial function [30]. However, all treatment delay groups were combined to help compensate for any potential power loss. Second, the original study focused on acute treatment efficacy; however, because Nf-L levels peak approximately 1 week after brain injury in pigs [24,49,69], later measurements may reveal a different treatment effect and optimal dose. Future studies should investigate longer-term efficacy of Cyclosporin A on serum biomarker concentrations. Third, while Quanterix originally developed the antibodies used in this study for human quantification, the assay has been successfully applied to swine previously and provides a translated assay for research [49,56]. Fourth, the current study focused on a limited set of protein biomarkers that have been used effectively in TBI, and neuropathology and mitochondrial function data was previously published [30]. Additional biofluid-based biomarkers such as inflammatory proteins or micro-RNAs, in which CsA accelerates downregulation of pro-inflammatory genes [77], or objective and behavioral/functional assessments, may provide effective treatment tracking targets for future studies. Fifth, only female piglets were included with sex differences expected to be negligible at the prepubescent stage studied; however, future studies should consider potential sex differences in treatment efficacy and including subjects of both sexes. Lastly, pediatric animals, pediatric humans, and adult humans are known to differ in baseline biomarker levels, so direct translatability by sex, age, and species cannot be assumed and should be investigated.

## 5. Conclusions

Focal and diffuse brain injury mechanisms resulted in distinct GFAP timelines and GFAP and Nf-L magnitudes. Focal CCI specifically had higher GFAP levels at 1 day than 1 h, indicating potential utility of GFAP evaluation beyond the currently approved 24 h window for CT scan recommendations. Cyclosporin A (10 and 40 mg/kg/day) reduced Nf-L levels at 1 day after diffuse TBI and did not change from baseline after focal TBI (40 mg/kg/day), showing promise of acute therapeutic benefit. Serum Nf-L levels peaked around 1-week post-injury, so further assessments of Nf-L at later timepoints may reveal greater or temporary therapeutic efficacy of acute treatment, warranting further investigation at extended timelines. Future porcine preclinical and clinical trials should consider utilizing the minimally invasive biomarker as an outcome metric along with measures of behavioral and physiological improvements for parallel designs to potentially improve the direct translatability of treatment efficacy measures.

## Figures and Tables

**Table 1:** Group sizes and number of serum samples at each timepoint.

Injury Type	Treatment		Group	Timepoint		
	Type	(mg/kg)	*n*	Pre	1 h	1 Day *
Sham	N/A	N/A	10	10	8	10
CCI	Saline	N/A	13	12	7	13
	CsA	10	10	10	5	10
		20	9	9	4	9
		40	9	9	4	9
		60	8	8	4	8
RNR	Saline	N/A	39	39	24	37
	CsA	10	29	29	0	29
		20	28	28	15	28
		40	27	27	0	27
		60	28	28	0	28

* 1 day samples were collected at 25 or 30 h post-injury depending on treatment delay.

## Data Availability

The original contributions presented in this study are included in the article. Further inquiries can be directed to the corresponding author, Susan Margulies.

## Abbreviations

ATP	Adenosine triphosphate
BBB	Blood–brain barrier
CV	Coefficient of variance
CT	Computed Tomography
CCI	Controlled cortical impact
CsA	Cyclosporin A
CSF	Cerebrospinal fluid
FDA	Food and Drug Administration
GFAP	Glial fibrillary acidic protein
IV	Intravenous
LOD	Level of detection
LLOQ	Lower limit of quantification
mPTP	Mitochondrial permeability transition pore
Nf-L	Neurofilament light
RNR	Rapid non-impact rotation
TBI	Traumatic Brain Injury
UCH-L1	Ubiquitin carboxyl-terminal hydrolase L1
ULOQ	Upper limit of quantification
